# The Use of Medical Crowdfunding to Mitigate the Personal Costs of Serious Chronic Illness: Scoping Review

**DOI:** 10.2196/44530

**Published:** 2023-12-04

**Authors:** Mary Killela, Caitlin Biddell, Jessica Keim-Malpass, Todd A Schwartz, Sandra Soto, Jessica Williams, Sheila Santacroce

**Affiliations:** 1 School of Nursing University of North Carolina at Chapel Hill Chapel Hill, NC United States; 2 Department of Health Policy and Management Gillings School of Global Public Health University of North Carolina at Chapel Hill Chapel Hill, NC United States; 3 School of Medicine University of Virginia Charlottesville, VA United States; 4 Department of Biostatistics Gillings School of Global Public Health University of North Carolina at Chapel Hill Chapel Hill, NC United States

**Keywords:** COVID-19, chronic illness, costs, financial, stress, appraisal, coping, crowdfunding, social network, social support, caregiver, systematic scoping review, medical crowdfunding, social network, coping behavior

## Abstract

**Background:**

Persons diagnosed with serious chronic illnesses and their caretakers experience multiple types of financial costs that strain their income and generate financial distress. Many turn to medical crowdfunding (MCF) to mitigate the harms of these costs on their health and quality of life.

**Objective:**

This scoping review aims to summarize the research on MCF for persons diagnosed with serious chronic illness regarding study designs and methods; the responsible conduct of research practices; and study foci as they relate to stress, stress appraisals, and the coping processes.

**Methods:**

This review was conducted in accordance with the PRISMA (Preferred Reporting Items for Systematic Reviews and Meta-Analyses) and PRISMA-ScR (Preferred Reporting Items for Systematic Reviews and Meta-Analyses extension for Scoping Reviews) guidelines. Eligible studies were conducted in countries designated as high income by the World Bank and focused on beneficiaries diagnosed with serious chronic illness. The findings of the included studies were summarized as they related to the key concepts in a conceptual framework derived from an established stress, appraisal, and coping framework and a conceptual model of financial toxicity in pediatric oncology.

**Results:**

Overall, 26 studies were eligible for inclusion in the review. The main findings included a lack of integration of qualitative and quantitative approaches and the inconsistent reporting of the responsible conduct of research practices. The included studies focused on financial stressors that contributed to financial burden, such as out-of-pocket payments of medical bills, basic living expenses, medical travel expenses, and lost income owing to illness-related work disruptions. Few studies addressed stress appraisals as threatening or the adequacy of available financial resources. When mentioned, appraisals related to the global financial struggle during the COVID-19 pandemic or the capacity of social network members to donate funds. The consequences of MCF included the receipt of 3 forms of social support (tangible, informational, and emotional), privacy loss, embarrassment, and the propagation of scientifically unsupported information. Studies found that friends and family tended to manage MCF campaigns. Although most of the studies (21/26, 81%) focused on monetary outcomes, a few (5/26, 19%) concentrated on peoples’ experiences with MCF.

**Conclusions:**

The identified methodological gaps highlight the need for more robust and reproducible approaches to using the copious data available on public MCF platforms. The integration of quantitative and qualitative methods will allow for nuanced explorations of the MCF experience. A more consistent elaboration of strategies to promote the responsible conduct of research is warranted to minimize risk to populations that are vulnerable and express concerns regarding the loss of privacy. Finally, an examination of the unanticipated consequences of MCF is critical for the development of future interventions to optimize existing supports while providing needed supports, financial and nonfinancial, that are lacking.

## Introduction

### Background

Medical crowdfunding (MCF) is the use of online platforms to raise funds to offset financial burden created by accumulating expenses and declining income for people experiencing health problems [1]. This financial coping behavior requires a person in need (beneficiary) or someone close to them (campaigner) to create a *campaign* that is then shared. Ideally, MCF involves not only the social network of the beneficiary but also strangers who come across the campaign providing details of the story of the beneficiary’s health and finances. Although MCF is often described as an opportunity to organize social networks to provide financial support, campaigners have varied experiences. Sometimes the campaign can *go viral* (ie, shared far and wide within a brief time period) and generate sizable financial support [2], whereas other campaigns receive scant attention and produce little to no financial support, including from friends and family [3].

The ultimate goal of MCF is to alleviate the beneficiary’s financial burden (financial demands on income and other assets with monetary value) and thus their financial distress (worry about money). Financial burden is a major psychosocial stressor with adverse effects on health and illnesses [4-7]. Serious chronic illnesses are health conditions that have been diagnosed by qualified health care professionals, require medical treatment and self-management for at least 3 months, and threaten life or quality of life [8]. Persons with serious chronic illness and their caregivers often need to take frequent or extended time away from work or school [9]. The work disruptions jeopardize their ability to maintain full-time employment and thus employer-sponsored health insurance coverage and enough income to pay for food, housing, and utilities. In addition, individuals with serious illness also have increased out-of-pocket costs, including for health-related travel [10]. Individuals with serious chronic illness have a great financial need, which aligns with the fact that a major MCF platform, GoFundMe, cites that more than one-third of all campaigns created on its platform fall into the medical category [11].

The practice of MCF has existed for a little more than a decade (GoFundMe was created in 2010). Consequently, the study of MCF is in its early stages [12]. Describing the methodologies used in this research area highlights how researchers are navigating MCF and exposes gaps in their current approaches. In addition, describing methodologies can elucidate strategies used to examine online talk, which is abundant on MCF pages. Online talk is any conversational text posted in a online space [12]. MCF sites include both online talk and campaign outcomes measured in the number of shares and amount of funds raised, respectively. Much of the current literature has also focused on success factors as they relate to campaign outcomes (ie, how much money has been raised). Some of these factors include title and narrative length, younger age, gender (men), and early position in search results [13,14]. Understanding both campaign outcomes and the content of online talk on MCF pages is critical to describing campaigners’ financial coping experiences and identifying the utility of this financial coping behavior.

Moreover, research on the application of new technology, such as the use of online platforms for MCF, requires considerations regarding the responsible conduct of research, and the current practices among studies examining MCF have not yet been summarized. Particularly in the case of MCF among individuals with serious chronic illness, there is a need for considerations of responsible conduct around privacy. There is currently a heterogeneity of considerations to protect the privacy of individuals’ health and financial status on MCF pages, and summarizing these is important to guide further work with these populations and data sources.

### Objectives

**Figure 1:**
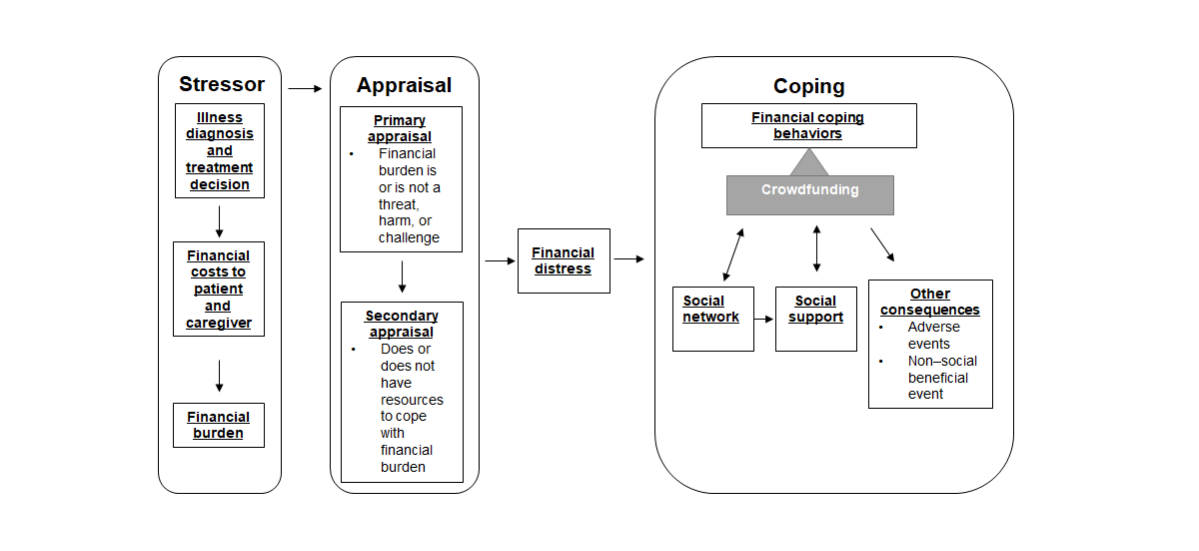
Framework of stress, appraisal, and coping in medical crowdfunding.

The purpose of this paper was to map the current literature of MCF for individuals with serious chronic illness and identify gaps as they relate to this scoping review’s conceptual framework. The conceptual framework that guided this scoping review derives from the stress, appraisal, and coping framework formulated by Lazarus and Folkman [15] as well as the model of financial toxicity in pediatric oncology developed by Santacroce and Kneipp [16] (Figure 1 [15,16]). The first framework links the financial coping behavior of MCF to the stressors and appraisals that precede it, and the second framework links serious chronic illness to the stress, appraisal, and coping framework. The model guiding this review begins with the cognizance of financial burden in serious chronic illness and initiates a primary appraisal of whether this psychosocial stressor threatens health and quality of life. Subsequently, threat appraisals trigger secondary appraisals to determine whether available resources are sufficient to cope with financial burden appraised as threatening. Serious chronic illness–related financial burden appraised as threatening and resources appraised as insufficient generate financial distress, followed by financial coping behaviors [16]. MCF is a financial coping behavior involving the solicitation of donations via an online platform by creating a web page that details the story of the person experiencing financial stress and asks social network members and other social media users to contribute funds. Sharing the story of a person’s health and finances in public online spaces to raise funds may have unanticipated beneficial as well as adverse consequences.

The review addresses the following three research questions: (1) What methods have been used to study MCF? (2) What actions were taken to ensure the responsible conduct of the research? (3) What descriptions of MCF, contributors to financial burden, stress appraisals, social network characteristics, social support exchanged, and other consequences of MCF were identified by prior studies?

## Methods

### Overview

This review was conducted in accordance with the PRISMA (Preferred Reporting Items for Systematic Reviews and Meta-Analyses) [17,18] and PRISMA-ScR (Preferred Reporting Items for Systematic Reviews and Meta-Analyses extension for Scoping Reviews) [19] guidelines. Search terms were related to crowdfunding and health, and search results were screened (title and abstract, followed by full text) to identify eligible articles. Once the sample had been established, findings from studies were extracted and then compiled into a series of matrices related to the research questions. The findings were then summarized and analyzed to see how they aligned with the conceptual framework guiding this review [20,21].

### Inclusion and Exclusion Criteria

The included studies focused on MCF by, or on behalf of, persons residing in high-income countries (HICs) and experiencing serious chronic illness, given that financial stressors inherent in long-lasting illnesses differ from those inherent in conditions that are time limited [22]. Serious chronic illness was defined in this instance to include illnesses that had been formally diagnosed, persisted for at least 3 months, and threatened life or quality of life [8]. Studies that used qualitative and quantitative approaches were included to comprehensively describe the approaches used. Studies that focused on crowdfunding to raise money for charitable foundations or research purposes or to assess the effects of crowdfunding on economic markets were excluded.

### Procedures

A health sciences librarian was consulted to create the strings of search terms and identify the computerized databases to be searched. The databases searched were PubMed, CINAHL, Embase, EconLit, and Scopus. The search was limited to English-language publications. No limits were placed on publication years. The search cast a wide net to identify published studies of crowdfunding and subsequently, through title and abstract screening, eliminate studies not focused on MCF in the context of serious chronic illness. Two reviewers (MK and CB) independently screened the titles and abstracts of studies identified through the database searches and subsequently read the full texts of studies that met the inclusion criteria. The reference lists of the included studies were scanned for studies not identified through the computerized searches [21]. The final search was conducted on February 16, 2022 (refer to Textbox 1 for the search strings).

**Strings of search terms created for this review.**

**Databases and search strings**

- PubMed, CINAHL, and Embase: “crowdfunding” or “crowdfund” or “crowdfunded”
- Scopus and EconLit: (TITLE-ABS-KEY (“crowdfunding” OR “crowdfund” OR “crowdfunded”)) AND (“medical” OR “health” OR “patient” OR “patients” OR “healthcare”)

### Data Collection and Management

Data from the included studies were collected using a standardized extraction tool derived from the conceptual framework (Multimedia Appendix 1). The tool was created to map on to the 3 research questions with basic information about the study; the methodology components of the study (informed by the concepts related to research on online talk [12]); the components of the responsible conduct of research mentioned in the Belmont Report, including respect for persons, beneficence, and justice [23]; and the components of the conceptual framework [15,16]. Specifically, concepts include information on the campaign beneficiary’s diagnosis and illness phase, stressors that led to MCF, stress appraisal, the beneficial and adverse consequences of MCF, and any other concepts that emerged from the included studies. Location was determined by country identified from MCF pages, not the country of the study team. The initial extraction tool was discussed between the 2 reviewers to identify areas for improvement. Regarding the responsible conduct of research, studies were examined to see whether the authors asked for consent or, in cases of publicly available data, made any modifications to protect the privacy of the person (respect for persons), as well as whether any steps were taken to protect the stored data (beneficence and justice). Upon completion of the tool, the second reviewer agreed that the tool adequately represented the information needed to address the research questions, and few clarification adjustments were made. Once the tool was finalized, the first reviewer independently conducted all extractions, whereas the second reviewer independently completed 20% of the extractions for quality assurance. Both reviewers met regularly to discuss consistency and resolve disagreements.

### Synthesis of Results

The extracted data were compiled in matrices to identify gaps and summarize key findings [24], including the counts of studies that addressed specific components of the study framework, used particular methodological approaches, or addressed the responsible conduct of research.

## Results

### Database Searches and Screening

**Figure 2:**
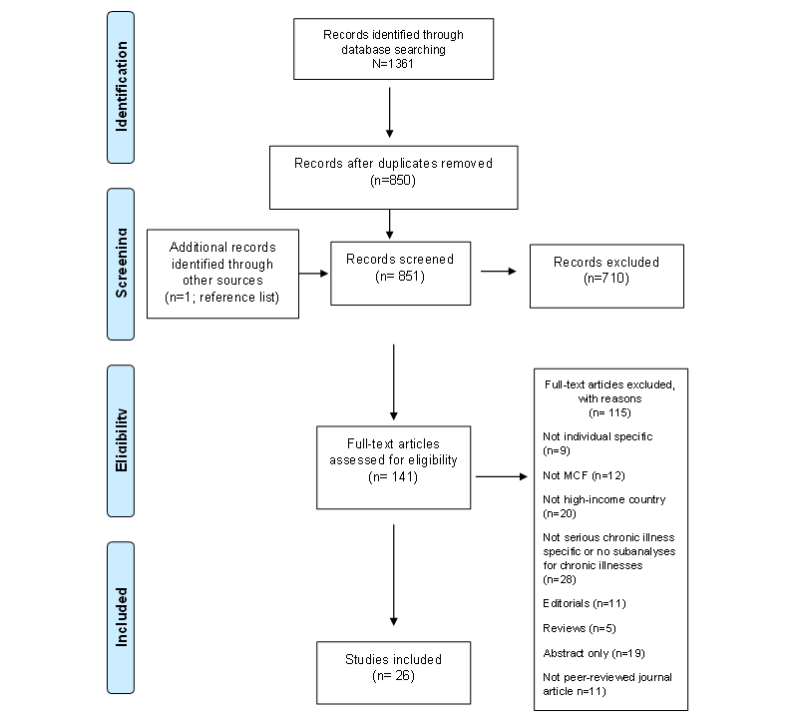
PRISMA (Preferred Reporting Items for Systematic Reviews and Meta-Analyses) diagram showing the number of studies identified, screened, assessed for eligibility, and included in the final analysis. MCF: medical crowdfunding.

The initial database searches yielded 850 unique studies (Figure 2 [18]); after screening, we excluded 824 (96.9%) studies, and the final sample consisted of 26 (3.1%) studies (Tables 1 and 2). There were 2 major reasons for the exclusion of studies: the beneficiary did not live in an HIC, and the beneficiary did not have a serious chronic illness. Publication years ranged from 2017 to 2022, with the majority published in 2020 and 2021 (16/26, 62%). Of the 26 studies, 10 (38%) [1,3,25-32] comprised campaigns located in the United States only, 6 (23%) [33-38] involved MCF campaigns located in the United States and other HICs, and another 6 (23%) [39-44] detailed MCF campaigns located in Canada. Other campaigns were located in Aotearoa New Zealand [45,46], Australia [36], France [37], Germany [37,47], Ireland [37], Belgium [48], and the United Kingdom [33,35,37]. Of the 26 studies, 2 (8%) [1,46] used theoretical frameworks (conceptualization of systems and biopower [49,50]) to guide their research.

**Table 1:** Key findings of studies that used quantitative methods or multiple methods.

Authors, year	Population studied (sample size)	Main findings related to stress, appraisal, and coping
Cohen et al [25], 2019	Cancer (1035)	Top reasons for medical crowdfunding: direct medical costs, travel costs, and nonmedical costs. Fundraising goals were much higher than funds raised, and there were large gaps in these ranges. The person who created the page was often a “third party” (nonself) campaigner.
Di Carlo et al [39], 2021	Urological conditions (benign and cancer conditions; 119)	Top reasons for medical crowdfunding: direct medical costs, lost income, nonconventional treatments, and travel. Most of the campaigns (87%) were created by friends or family.
Ho et al [27], 2019	Cancer (143)	Top reasons for medical crowdfunding: direct medical costs, travel, housing and living expenses, and replacement of lost income. The average goal amount was approximately 4 times the average amount of funds raised. The 3 campaigns that raised the most money accounted for more than half of all the funds raised in the study sample. Approximately a quarter of the campaigns included education on CAR^a^ T-cell therapies.
Holler et al [28], 2022	Urological cancers (1234)	Top reasons for medical crowdfunding: direct medical costs and nonmedical costs. Kidney cancers were the most common, yet testicular cancers raised the most amount of funds. Most of the campaigns were not authored by the beneficiary, and most of the beneficiaries had advanced-stage cancer.
Kenworthy et al [1], 2020	Medical costs and health care costs (637)	Among the campaigns that were created by the beneficiary, 67% were created by women; among the campaigns that were created by a friend or family member, approximately 80% were created by women. There are racial disparities in medical crowdfunding, both in terms of presence on the campaign websites (only 8% of the campaigns were on behalf of a Black beneficiary, and only 10% of the campaigns were on behalf of beneficiaries who were non-Black persons of color) and in terms of the amount of funds raised (Black campaigners raised on average US $22 less per donation).
Livingstone et al [48], 2021	Spinal muscular atrophy (171)	Medical crowdfunding efforts raising funds for drugs for rare diseases have astronomically high goals to cover the cost of treatment. Often, this is not possible through an individual’s preexisting social networks. Campaigns often included instances of minimizing the risks of treatments.
Loeb et al [33], 2018	Cancer (400)	Top reasons for medical crowdfunding: direct medical costs and lost income. Lost income was present in 4.5% of the prostate cancer campaigns on GoFundMe, 17% of the prostate cancer campaigns on YouCaring, and 2% of the breast cancer campaigns on GoFundMe.
Lublóy [47], 2020	Cancer (101), mental disorder (34), disability (26), accidents (23), lipoedema (22), genetic disorders and rare diseases (20), care of older adults and people with dementia (19), multiple sclerosis (15), and oral health (15)	Top reasons for medical crowdfunding: therapy (often alternative, scientifically unsupported therapies), living expenses, and treatment-related costs. The diagnoses that had the highest funding goals were care of older adults and people with dementia, transplants, and cancer.
Pol et al [41], 2019	Kidney and liver transplants (429)	Top reasons for medical crowdfunding: living expenses, relocation, travel expenses, and loss of income. More campaigns in this sample were for kidney transplants than for liver transplants. Prayers were the most commonly requested form of nonfinancial support.
Prabhu et al [34], 2021	Cancer (555)	Top reasons for medical crowdfunding: travel and living expenses. Some campaigns provided explanations of the therapies or links to provide their donors with more information. Proton therapy was the radiation type with the highest funds raised and highest goals set.
Silver et al [29], 2020	Cancer (144,061)	Top reasons for medical crowdfunding: direct costs or loss of income related to cancer treatment. The findings indicated that most of the campaigns originated from counties with low neighborhood deprivation index scores. Furthermore, counties with higher neighborhood deprivation index scores (lower-resourced counties) raised less money.
Snyder et al [30], 2020	Cancer, autism spectrum disorder, Niemann-Pick disease type C, amyotrophic lateral sclerosis, kidney failure, and muscular dystrophy (53)	The descriptions of what funds were needed for were similar for both expanded access campaigns and right-to-try campaigns, including direct medical costs and travel costs. An exception was that some sponsors in the expanded access pathway covered the direct costs of treatment.
Snyder et al [35], 2020	Cancer (1309)	Top reasons for medical crowdfunding: supplements (30.2%), better nutrition (21%), and high-dose vitamin C (19.8%). Beneficiaries with late-stage cancers (stage IV: 55.8%) made up the majority of the sample using medical crowdfunding to fund CAM^b^ interventions.
Song et al [32], 2020	Cancer (500)	Higher-staged cancer was more present in CAM campaigns than in non-CAM campaigns (54% vs 12%). Medical crowdfunding is a tool to raise funds for individuals who prefer to attempt to treat cancer with CAM methods rather than with conventional cancer treatment; medical crowdfunding platforms can also be used to spread scientifically unsupported information.
Thomas et al [36], 2021	Kidney cancer (486)	Top reasons for medical crowdfunding: direct medical costs, nonmedical costs, travel expenses, and lost income. Among the survivors of cancer, 37% were primary wage earners, 43% reported having to greatly decrease their work hours owing to their illness, and 34.4% mentioned that the beneficiary was employed. Most of the campaigners were friends (28.8%) and family (14.4%). Approximately 8% of the campaigns mentioned an offline fundraiser (eg, a gala or a community dinner).
van Duynhoven et al [43], 2019	Cancer (1788)	Campaigns that were created to address the costs of cancer treatment in Canada were found to be primarily among individuals who live in areas that are high income, are well educated, have high percentages of home ownership, and live near city centers.

^a^CAR: chimeric antigen receptor.

^b^CAM: complementary and alternative medicine.

**Table 2:** Key findings of qualitative studies.

Authors, year	Population studied (sample size)	Main findings related to stress, appraisal, and coping
Ghazal et al [26], 2022	Cancer (46)	Medical crowdfunding among adolescent and young adult survivors of cancer illuminated the tension between needing funds and being appreciative of received funds. Adolescent and young adult survivors of cancer also felt humiliated to ask for funds and share personal and vulnerable details about their life and illness.
Kenworthy [3], 2021	Type 1 diabetes (2)	Top reasons for medical crowdfunding: direct medical costs and lost income. Beneficiaries masked financial struggles to convince their donors that their donation was influential. Beneficiaries experienced comparative suffering and self-blame when campaigns were not monetarily successful.
Neuwelt-Kearns et al [45], 2021	Cancer (8), motor neuron disease (1), multiple sclerosis (2), cerebral palsy (1), anorexia nervosa (1), autoimmune disease (1), and paraplegia (1)	Campaigners felt that campaigns needed to capture the attention of the crowd, demonstrate deservingness, and be accountable to the donors to continue to demonstrate their worth.
Palad and Snyder [40], 2019	Drug and alcohol addiction (other addictions: food, smoking, gambling, sex, and hoarding; 129)	Top reasons for medical crowdfunding: to afford treatment (direct medical costs), to survive treatment (living expenses), and to start life anew after treatment. Beneficiaries were uncomfortable with discussing private information but felt it necessary to receive the help that they needed.
Snyder et al [42], 2017	Cancer, kidney disease, neurological disease, and Lyme disease (80)	Top reasons for medical crowdfunding: hospital parking, travel expenses, living expenses, time off work (lost income), experimental interventions, and direct medical costs not covered by the Canadian public health system. Requests indicated that beneficiaries asked for financial donations as well as love and support from family and friends.
Snyder and Turner [31], 2018	Multiple sclerosis, chronic obstructive pulmonary disease, disease of the eye, joint disease, and Parkinson disease (78)	Top reason for medical crowdfunding: direct medical costs. The persons involved used research as a way to communicate credibility and also to communicate that these interventions were not covered by insurance because they were experimental and that by participating they were helping the next generation by taking part in research on unproven stem cell–based interventions.
Vassell et al [44], 2020	Lyme disease (238)	Top reasons for medical crowdfunding: direct costs (holistic treatments, experimental stem cell therapies, and antibiotic treatments), living expenses, and travel costs (parking and car maintenance). Some narratives of individuals with Lyme disease included a discussion of problems with the Canadian public health system and advocacy for better awareness of, and treatment for, Lyme disease.
Wardell [46], 2021	Cancer (23) and unspecified (36)	The presentation of need during the COVID-19 lockdown in Aotearoa New Zealand changed the way campaigners were communicating their illness story. It was important for campaigners to express their need in the context of COVID-19, which had the dual impact of creating a shared experience with their social network while also explaining that their need was still great.
Zenone et al [37], 2020	Cancer (155)	Top reason for medical crowdfunding: cost of purchasing CBD^a^ products. Among the campaigns, 45.8% were for individuals with stage IV or terminal cancer. Some of the campaigns contained anecdotal evidence suggesting that CBD was efficacious in ways that are not evidence based or misinterpreted available information.
Zenone et al [38], 2021	Cancer (96), seizure-inducing diseases or conditions (48), other or unspecified (6), joint or inflammatory diseases (6), mental health disorders (3), nervous system diseases (3), and autoimmune disease (2)	This study focused on informational pathways that led campaigners to pursue CBD as a curative or palliative treatment. The analysis identified that many campaigners found CBD through their own research, with the minority using the recommendation of a trusted health care provider. Campaigns were both sources of scientifically unsupported information and informational support.

^a^CBD: cannabidiol.

### Methodological Approaches Used

Of the 26 studies, 10 (38%) [3,26,31,37,38,40,42,44-46] used qualitative methods, 12 (46%) [1,25,27-29,32-35,39,43,47] used quantitative methods, and 4 (15%) [30,36,41,48] used multiple methods. The sample sizes in all included studies ranged from 2 to 144,061 (median 204.5, IQR 89.8-541.3); 19 (73%) of the 26 studies had sample sizes of ≥100. Among the qualitative studies, sample sizes ranged from 2 to 238 (median 79, IQR 49.3-148.5), whereas among the quantitative studies, sample sizes ranged from 119 to 144,061 (median 596, IQR 395-1,252.8). The studies that used multiple methods had sample sizes ranging from 53 to 486 (median 300, IQR 141.5-443.3).

Most of the included studies (20/26, 77%) [1,25,27-41,46-48] used data from publicly available MCF pages. These studies analyzed either the narrative description written by the campaigner (a component of online talk; 20/20, 100%) [1,25,27-41,46-48] or reported campaign outcomes (eg, the goal amount of funds, the number of social media shares, and the amount of funds raised; 19/20, 95%) [1,25,27-41,47,48]. Of the 26 studies, 1 (4%) [43] analyzed campaign outcomes alongside data from other sources such as census data, 2 (8%) [3,45] conducted interviews with MCF beneficiaries and campaigners, and 1 (4%) [26] used web-based methods to survey beneficiaries about their experiences with MCF.

### Campaign Characteristics

All included studies that used data available on MCF pages bounded their data (20/26, 77%). *Bounding* refers to setting limits on the data that will be examined [12], typically either to enhance the feasibility of the research or to focus on the population of interest. Most of the included studies were bounded by MCF platform or website (24/26, 92%) [1,25,27-48]; the clinical characteristics of campaign beneficiaries, such as person-reported medical diagnosis (20/26, 77%) [25-41,43,44,48]; and when the campaign platform was searched to obtain a sample (6/26, 23%) [30,35,37,38,43,46]. MCF platforms included GoFundMe (22/26, 85%), YouCaring (5/26, 19%), FundRazr (2/26, 8%), JustGiving (1/26, 4%), Generosity (1/26, 4%), Leetchi (1/26, 4%), and Givealittle (2/26, 8%).

Most of the studies (24/26, 92%) collected data from publicly available MCF campaign pages, and the campaign was the unit of analysis. Exceptions include 12% (3/26) [3,26,45] of the studies, which used primary data collection with the individual as the unit of analysis. Analysis of data from publicly available MCF campaign pages involved transforming the extracted data into a usable format for analysis. Of the 26 studies, 3 (12%) [28,32,36] were analyses of a parent data set created for another study included in this scoping review [25]. In other words, 4 (15%) of the 26 studies were drawn from the same data set of cancer crowdfunding campaigns. All included studies were retrospective and cross-sectional (26/26, 100%). Most of the studies (22/26, 85%) specified the date on which the MCF platform was searched for eligible campaigns to create a study sample. For the quantitative studies, the researchers did not explicate whether they extracted all data (eg, narratives and updates) posted between the campaign launch date and the sampling date or only the most recently posted data.

### Strategies to Ensure the Responsible Conduct of Research

Of the 26 studies, 5 (19%) [25,26,29,36,41] explicitly stated that the study was approved by an institutional review board (IRB) and exempted from further review. The reasons for exemption included the following: data were publicly available, data were deidentified, the researchers had no direct contact with participants, and the study was deemed as not involving human participants. Of the 26 studies, 11 (42%) [27,30,32,33,37,38,40,42,44,47,48] did not mention communicating with an IRB about the study, whereas 10 (38%) [1,3,28,31,34,35,39,43,45,46] mentioned communication with their IRB but were unclear about the nature of the review (expedited or full) and the requirements for continuing the review. Of the 26 studies, 8 (31%) [1,3,26,36,38,43,45,46] elaborated on strategies to minimize risks to individuals’ privacy and the confidentiality of their data. Such strategies included deidentifying data, storing data on password-protected encrypted drives, reporting results in summary form, changing or removing quotations so that identities were not easily discoverable through search engines, and using pseudonyms in the data and reports of study results.

### Stress, Appraisal, and Coping Concepts Studied

Key findings of the included studies are shown in Table 1 (studies that used quantitative or multiple methods) and Table 2 (studies that used qualitative methods).

### Sample Characteristics

Of the 26 studies, 19 (73%) either focused on MCF to raise funds for beneficiaries with cancer (n=12, 63%) [25-29,32-37,43] or included MCF for beneficiaries with cancer in the sample (n=7, 37%) [30,38,39,42,45-47]. Illness was described in terms of disease stage or illness phase compared with active treatment. Among the studies that reported the beneficiaries’ disease stage (6/26, 23%) [25,30,32,35,37,38], stage IV or end-stage disease was reported most often and applied to between 23.1% and 55.8% of the sample (4/6, 67%) [25,30,35,37]. For the studies that reported the beneficiaries’ illness phase (3/12, 25%) [26,28,34], active treatment predominated. Tables 1 and 2 present information about the populations represented in the samples of the included studies.

Given that the unit of analysis in the included studies was usually the campaign, participant demographics were typically unclear; for instance, campaigns consist of campaigners and beneficiaries—the campaigner and the beneficiary may or may not be the same person. Some of the studies reported demographics (eg, biological sex, gender, race, and ethnicity) for beneficiaries only (5/26, 19%) [3,26-28,34,36] or campaigners only (1/26, 4%) [45]. Others reported demographics for beneficiaries and campaigners (1/26, 4%) [1] or were unclear about whose demographics were being reported (2/26, 8%) [25,32].

Of the 26 studies, 11 (26%) [1,3,25-28,30,34,36,41,45] reported beneficiary age. In 10 (91%) [1,3,25-28,34,36,41,45] of these 11 studies, at least 70% of the beneficiaries were adults. Of the 26 studies, 10 (38%) [1,25,26,28,33,34,36,39,40,45] reported the relationship between campaigners and beneficiaries. The most commonly reported types of relationships were friends or family members (eg, the beneficiary’s child, spouse, parent, other immediate family member, other family, distant relative, friend, or coworker). Rarely was the campaigner also the beneficiary.

Most of the studies (21/26, 81%) described MCF in terms of funds raised per campaign and median or average total funds raised across campaigns in the sample. However, the amounts were often reported without much context provided about how these numbers related to the need of beneficiaries. Great variability was seen in campaigns’ monetary goals and funds raised. Other commonly reported campaign outcomes included the proportion of monetary goal achieved, the number of social media shares, the number of donors, the number of updates posted, and campaign duration. The included studies also identified the factors that influenced the extent to which the campaign was monetarily successful in terms of total funds raised and in comparison with the stated monetary goal. These factors included beneficiary demographics, diagnosis (usually cancer), the number of donors, the number of social media shares, high or low monetary goals, attitudes conveyed in the narrative, the type of treatment sought, and the divulgence of personal information about the beneficiary.

### Contributors to Financial Burden or Financial Stressors

Across studies, financial burden as a stressor was often a focus of the research. Most of the studies (24/26, 92%) [3,25-42,44-48] mentioned out-of-pocket expenses as a contributor to financial burden. These expenses included nonreimbursable payments for goods and services to treat their illness or manage symptoms. In 6 (25%) of these 24 studies, funds were needed to pay for specifically cannabidiol products [37,38], for therapies falsely marketed as curative options [48], for therapies not covered by insurance but accessible under expanded access or right-to-try legislation [30], or for an array of complementary and alternative medicine interventions [32,35]. Other contributors to financial burden included the usual expenses of daily living (eg, payments for housing, food, clothing, transportation, and utilities; 13/26, 50%) [3,25,26,28,34,36,40-42,44-47] and treatment-related travel and lodging (14/26, 54%) [25-27,29,30, 34,36,39,41,42,44-47]. Another contributor cited was lost income (13/26, 50%) [3,25,27,29,33,34,36,39-42,44,46] owing to illness-related employment disruptions for patients and their caregivers.

### Stress Appraisals

Of the 26 studies, 2 (8%) mentioned stress appraisals. The first study examined MCF during the COVID-19 lockdown in Aotearoa New Zealand. The authors discussed the primary appraisal of campaigners reflected in their narratives. Campaigners explained their appraisal of the global health crisis to donors and how the global health crisis exacerbated the beneficiary’s already tenuous personal situation [46]. The second study examined secondary appraisal, asking adolescent and young adult survivors of cancer about MCF. Participants communicated about whether they could access resources within their social networks to cope with the threat of financial harm. Some of the participants indicated that resources were available based on their socioeconomic status and the nature of their social networks, whereas others indicated that they lacked resources given their age and the financial circumstances of their friends and family [26].

### Consequences of MCF

Of the 26 studies, 12 (46%) [26,28,29,31,34,37,38,40,44-46,48] reported the consequences of MCF. Of these 12 studies, 4 (33%) [31,37,38,48] discussed that MCF platforms were used to spread information that was not necessarily scientifically supported. This information ranged from information about generally accepted complementary therapies for symptom management to using those same generally accepted complementary therapies outside of their generally accepted use (eg, in lieu of conventional cancer treatment). Examples include campaigns seeking to raise funds for (1) untested stem cell treatments in direct-to-consumer formats [31] and (2) cannabidiol products for curative purposes [37,38] or (3) overemphasizing the potential of a spinal muscular atrophy treatment in the absence of evidence to support its efficacy [48]. By contrast, other studies found that MCF platforms were used to spread awareness about certain illnesses (eg signs and symptoms) and for advocacy [27,44].

Other consequences were related to the experience of participating in MCF. These consequences included beneficiaries and campaigners feeling humiliated or embarrassed about MCF [26]. In addition, adolescent and young adult survivors of cancer explained that there were trade-offs between feeling humiliated and benefiting from the funds raised [26]. Such trade-offs included a loss of beneficiary privacy [26,40,45] and tensions regarding disclosing what is necessary to secure funds without divulging too much. Campaigners also described feeling pressured to maintain their campaign, communicate the beneficiaries’ deservingness to receive funds, and show fiscal accountability to donors while maintaining some modicum of privacy for the beneficiaries [45]. Finally, a consequence of the narratives written by campaigners during the COVID-19 lockdown was that they facilitated empathy between beneficiaries and donors. Campaigners achieved this by explaining the beneficiary’s financial struggle within the shared context of the COVID-19 pandemic health crisis [46].

### Social Support Exchanged and Social Networks Studied

Social support was primarily exchanged via the donation of funds (ie, tangible support). This tangible support often came from people with whom the campaigner or beneficiary had preexisting relationships; 1 (4%) [45] of the 26 studies indicated that strangers rarely offered this form of social support. However, the included studies found evidence of informational and emotional support in online talk. Of the 26 studies, 2 (8%) [27,44] mentioned that the campaigns provided informational support to the social networks of the beneficiaries by generating awareness regarding their illness and its trajectory and providing information about a lesser-known treatment option. The instances of informational support also included education about chimeric antigen receptor (CAR) T-cell therapy, radiation therapy [27,34], and sharing tips on surviving or thriving in isolation during the COVID-19 pandemic [46].

Emotional support exchanges included the sharing of well wishes and prayers [41,42]. References were made to social support provided offline [36], specifically events where funds were raised, and other forms of social support were likely exchanged. Another study found that social support, including tangible support in the form of monetary donations, diminishes over time, and that MCF pages were most active closest to campaign creation [26]. This same study indicated that MCF pages were helpful to the beneficiary’s social network members in terms of providing them with direction about what they might do to help when knowing what to do can be difficult [26].

## Discussion

### Principal Findings

This scoping review identified major gaps in the research, including a lack of information about the responsible conduct of research, little examination of stress appraisals, and little focus on the unanticipated consequences of engaging in MCF. Although the included studies used both qualitative and quantitative approaches, the results were not integrated to advance the understanding of individual experiences with MCF.

Although quantitative description can illuminate overall trends and patterns in MCF as a financial coping behavior, the unique value of an MCF campaign stems from the rich qualitative data that exist alongside the quantitative data. This review found that narratives were often used as the basis for additional statistics; for example, the authors may have examined the narratives for a discussion of the purpose of the campaign and then provided descriptive statistics on the different types of purposes. In other words, there were many across-case analyses but few within-case analyses. A gap remains in relating campaign narratives and campaign outcomes to the experience of the stressors that contribute to financial burden, the associated financial distress, and subsequent financial coping behaviors in the context of serious chronic illness. An examination of campaign narratives and the light they shed is key to informing clinicians and researchers about the potential financial consequences of the patient’s illness journey and identifying areas and inflection points for intervention.

Information about the responsible conduct of research was consistently lacking across the included studies. Guidance found in this review to address the responsible conduct of research included using pseudonyms, modifying quotes when possible, and summarizing results to control the minimal risks associated with the research [1,3,38,43,45,46]. However, these strategies were not consistently used across studies. Given that many studies identified concerns about trade-offs between sharing personal information and needing to raise funds, researchers of MCF should be cognizant of this vulnerability and use these strategies to minimize risks to beneficiary privacy in relation to data about their health and finances. Valdez and Keim-Malpass [51] provide a framework for researchers to use when making decisions about the appropriate responsible conduct of research on social media; for instance, in cases of public social media spaces, informed consent may not be possible if the researcher is unable to contact everyone represented in the online talk. Moreover, attempting to obtain consent may result in collecting more information than is necessary for the research and inadvertently threaten privacy [51]. In addition, the risks posed by the research are likely no greater than those encountered in everyday use of public social media spaces. However, authors still have the capability to respect the privacy of individuals posting in these spaces. Researchers must consult with their local IRBs and not proceed with the research until the study has been reviewed and approved. Perhaps even more influential would be the creation of consensus-based reporting guidelines for researchers using MCF pages as their data source. These guidelines could assist both IRBs and researchers in determining how best to maintain privacy and standardize approaches to the responsible research of MCF.

Although many of the studies (24/26, 92%) addressed the financial stressors that contributed to financial burden, social support exchanges, or other consequences of MCF (12/26, 46%), very few studies (2/26, 8%) explicitly addressed the primary and secondary appraisals of stressors. In this review, 1 (4%) of the 26 included studies described the primary appraisal process as comparing suffering, which resembles the cognitive coping strategy referred to as downward (or upward) comparison [52]. In downward comparison, individuals compare themselves with someone they see as less fortunate to boost their subjective well-being [52] or gain perspective. Evidence of comparisons regarding suffering, deservingness, or financial responsibility in MCF campaign online talk deserves further study and could be a common feature among campaigns, especially during the COVID-19 pandemic when a larger pool of people may be competing for a smaller pool of funds.

The appraisals of financial burden as threatening or not and other psychosocial stressors by persons living with serious chronic illness have likely been influenced by the COVID-19 pandemic even if this has been mentioned specifically in only 1 (4%) [46] of the 26 included studies. The COVID-19 pandemic has influenced individuals’ ability to work, especially in the service sector [53]; complicated the availability of childcare [54] and schooling; and generated additional out-of-pocket expenses (eg, personal protective equipment and at-home tests for COVID-19). The other study examining appraisal focused on the secondary appraisal of the adequacy of available financial resources [26]. Other studies examining MCF have also found that social capital (ie, wealth and other resources of one’s social network) is a critical factor in MCF that contributes to existing health disparities [3,55]. Knowing more about what resources individuals perceive as being available to them is instrumental in the development of nonredundant interventions that optimize the types of supports that individuals have or can access while adding what they do not have.

**Figure 3:**
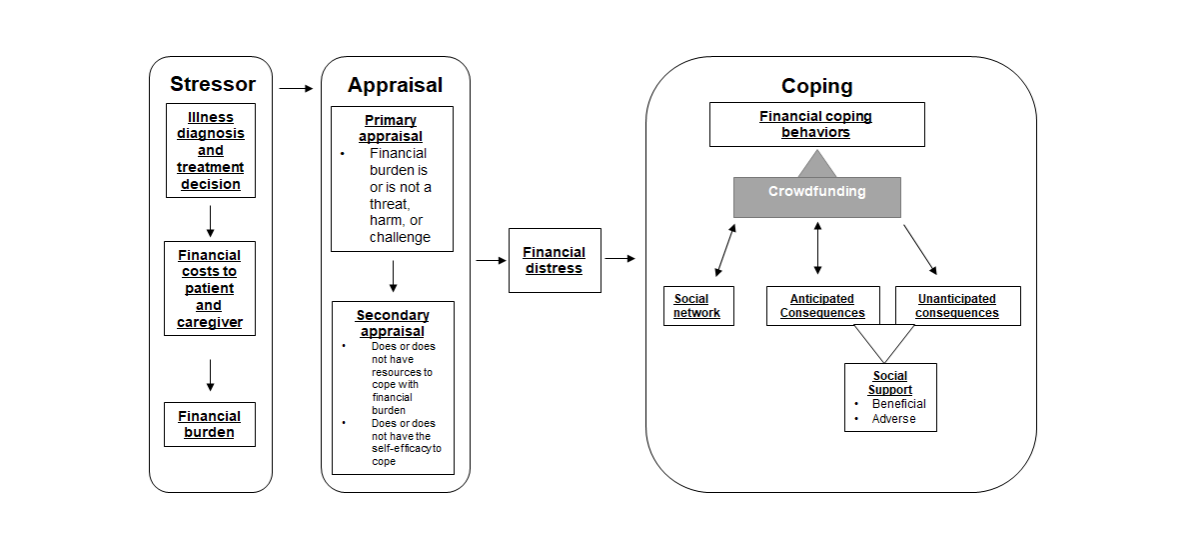
Refined conceptual framework of stress, appraisal, and coping in medical crowdfunding.

Many of the included papers focused on anticipated consequences, both beneficial and adverse. Conversely, few articles mentioned, and even fewer focused on, unanticipated consequences. Therefore, the conceptual framework was refined to specify the anticipated and unanticipated consequences of MCF (Figure 3). Highlighting what makes someone monetarily successful at MCF (an anticipated consequence) ignores both the systemic problems contributing to financial need and the other nonmonetary benefits (unanticipated consequences) of engagement in online communities [55-57]. The monetary benefits, although important and a major driver of engagement in MCF, may not be the only driver nor the only benefit. The importance of broadening the focus to include both anticipated and unanticipated consequences lies in the fact that it brings attention to the adverse effects of unmet needs and the beneficial effects of broadening one’s online social networks and thus the exchanges of other types of needed social support [55,57]. The receipt of funds, although important, may not be the only driver of participation in MCF. Therefore, the conceptual framework should reflect these varied consequences of MCF.

The unanticipated consequences found in this review leaned toward both beneficial and adverse consequences. The adverse unanticipated consequences included both loss of privacy and the spread of scientifically unsupported information. Of the 26 included studies, 1 (4%) discussed the trade-off between campaigners respecting beneficiary privacy and feeling a need to divulge personal information to convince donors that the campaign is legitimate and the beneficiary worthy [40]. Consistent with the findings of this review, other studies have found that people balance their financial needs with the personal medical information they are willing to share [58]. Scientifically unsupported information has also been highlighted in prior work. This, in contrast to fraud, seems to be in good faith, meaning that the beneficiary believes in or perhaps has hopes for the approach, although ill-informed or unfounded. This finding, along with contemporary conversations about trusting science and the propagation of scientifically unsupported information related to COVID-19 [59], indicates that MCF pages, similar to other forms of social media, have the potential to be dangerous. In addition, studies in this review identified concerns with regard to health equity in MCF campaigns. Several studies (3/26, 12%) found differences in the amount of money raised and the prevalence of campaigns by race, sex, neighborhood deprivation, income, and education [1,29,43], suggesting that MCF may exacerbate existing disparities in financial burden. As our review focused on individual-level stress, appraisal, and coping, we did not focus on the more systemic health equity implications of MCF. Nevertheless, this emerged as a notable component of the literature that merits future attention. Therefore, researchers ought to be mindful of health equity concerns regarding MCF, the privacy concerns of campaigners, and the reach and influence of information spread through MCF pages.

Some unanticipated consequences could also be perceived as benefits. These benefits include sharing illness journeys and receiving emotional support. A gap among current studies concerns the communication of beneficiaries’ illness journey and the factors that led to engaging in MCF. The literature to date has paid greater attention to beneficiaries’ deservingness and the direness of their situation [55,57]. However, prior studies have demonstrated that individuals feel a catharsis from writing about, and sharing, their experience and appreciate having a record of their illness journey to reflect about their growth over time [56]. Another benefit is the receipt of emotional support from online communities and having a space for illness-related informational exchanges [26,27,41,42]. MCF beneficiaries were extremely grateful for both monetary and nonmonetary support from their social network. Prior works also indicate that online social network members provided social support in non-MCF forums (eg, meal trains) and by commenting on, and sharing, MCF campaigns [5]. Furthermore, MCF pages served as hubs for communicating current and urgent needs in a one-to-many format [5]. Therefore, emotional support and sharing illness journeys highlight the utility of MCF that exists beyond the anticipated monetary support received.

There were limitations to this scoping review. First, a major focus of this review was to describe the topics of online talk as they relate to sections of the conceptual model. Therefore, this review did not dive into the concepts of marketing and financial success of MCF or relate the results to this literature. Second, the review is limited to English-language publications about MCF campaigns by individuals with serious chronic illness living in HICs. However, MCF exists and is important in countries that are not designated as high income by the World Bank. Future research should fill this gap by reviewing the research of MCF in countries with mid- or low-income economies. By contrast, the lack of reliable internet or broadband and other essential infrastructure, personal digital devices, and technical know-how likely thwart MCF in mid- and low-income countries where treatment abandonment (failure to start or complete effective therapy) owing to cost is a major contributor to excess mortality, especially for young female individuals living in poverty in rural areas and lacking social support networks [60,61]. Many studies identified through computerized database searches were excluded because they lacked subanalyses specific to serious chronic illness or did not determine where campaigners or beneficiaries were living or seeking treatment. Therefore, the findings of this review are limited in scope and not broadly generalizable. In addition, this review did not publish its protocol publicly; future work should adhere to this element of the PRISMA-ScR guidelines [19]. Finally, given the heterogeneity of designs and methodologies, comparisons across the included studies regarding financial outcomes are problematic. Establishing guidelines regarding MCF research methods will help future scholars to compare findings across studies.

### Conclusions

Although the use of social media platforms to raise needed funds is relatively new, this financial coping behavior for individuals with serious chronic illnesses is used by individuals in need. This review has identified gaps in the methodological approaches used to study MCF and in the foci of the research to date. Future research on MCF should qualitatively analyze more comprehensive stories of people’s journey to MCF and both anticipated and unanticipated consequences of engaging in this behavior so that we can design and adapt financial toxicity interventions to optimize the existing streams of support and supplement where support is lacking.

## Appendix

Multimedia Appendix 1 Extraction tool template.

Multimedia Appendix 2 PRISMA ScR Checklist.

## Abbreviations

CAR: chimeric antigen receptor
HIC: high-income country
IRB: institutional review board
MCF: medical crowdfunding
PRISMA: Preferred Reporting Items for Systematic Reviews and Meta-Analyses
PRISMA-ScR: Preferred Reporting Items for Systematic Reviews and Meta-Analyses extension for Scoping Reviews
