# Supplementary material for: The Use of Medical Crowdfunding to Mitigate the Personal Costs of Serious Chronic Illness: Scoping Review
Source: J Med Internet Res. 2023 Dec 4;25:e44530. doi: 10.2196/44530 (PMC10697184; doi:10.2196/44530)
Supplement: Multimedia Appendix 1 [file jmir_v25i1e44530_app1.pdf]

Extraction tool template

Reviewer:	
Author(s)/Year:	
Title	
Author contact information:	
Country:	
Purpose:	
Conceptual framework/ theoretical lens	

Methodology components

Approach	Qualitative	Quantitative	Multiple methods	Mixed methods	
Name of design (e.g., observational, descriptive, experimental, etc.)					
Online talk analyzed (narratives, updates, comments, shares, etc.)	Narrative/	Updates	Comments	Shares	Etc.
# OF CODERS					
Variables					
Eligibility criteria	INCLUSION		EXCLUSION		
Sampling method (purposive sampling, random sampling, etc.)					
Recruitment					
Bounded data: platform	GoFundMe	Facebook	Other-		
Bounded data: # of posts analyzed					
Sample size (n=?)			Unit of analysis		
			# of posts analyzed	# of campaigns analyzed	# of participants analyzed
Bounded data: # of months/years analyzed					
Representativeness of sample/contextual importance of sample					
Primary vs. secondary analysis	Primary data		Secondary data		
Publicly available	Publicly available		Not publicly available		

Longitudinal/cross-sectional	Longitudinal	Cross-sectional		
Retrospective/prospective	Retrospective	Prospective		
Data collection/procedure				
Analytical approach				
Quantification of qualitative data analytical approach (if applicable)				
Responsible conduct of research (respect for persons, beneficence, justice)	Respect for persons:	Beneficence:	Justice:	Not mentioned

Framework components

	EVIDENCE	OTHER COMMENTS
Description of the population (e.g., diagnosis, age, baseline condition/illness trajectory.) (Bounded by people):		
Stressors (Antecedents to crowdfunding)		
Appraisal<br>- primary<br>- secondary		
Descriptions of crowdfunding		
Consequences of crowdfunding		
Social network characteristics		
Social support		
Other key concepts studied		
Emergent concepts		
